# A farewell to phlebotomy—use of placenta-derived drugs Laennec and Porcine for improving hereditary hemochromatosis without phlebotomy: a case report

**DOI:** 10.1186/s13256-021-03230-5

**Published:** 2022-01-23

**Authors:** Yuki Hamada, Eiichi Hirano, Koji Sugimoto, Keizo Hanada, Taiichi Kaku, Naoki Manda, Kenichi Tsuchida

**Affiliations:** 1 Hamada Clinic for Gastroenterology and Hepatology, Sapporo, Japan; 2 Research Institute, Japan Bio Products Co., Ltd., 1-1 Kurume Research Center bldg. 2F, Hyakunenkoen, Kurume, Fukuoka 839-0864 Japan; 3 Japan Bio Products Co., Ltd., Tokyo, Japan; 4 Manda Memorial Hospital, Sapporo, Japan

**Keywords:** Hereditary hemochromatosis, Phlebotomy, Human placental extract, Hepcidin, Hepatocyte, Iron metabolism, Iron-overloading disease, Case report

## Abstract

**Background:**

Human hepcidin, produced by hepatocytes, regulates intestinal iron absorption, iron recycling by macrophages, and iron release from hepatic storage. Recent studies indicate that hepcidin deficiency is the underlying cause of the most known form of hereditary hemochromatosis.

**Case presentation:**

A 44-year-old Asian man who developed type 2 diabetes mellitus had elevated serum ferritin levels (10,191 ng/mL). Liver biopsy revealed remarkable iron deposition in the hepatocytes and relatively advanced fibrosis (F3). Chromosomal analysis confirmed the presence of transferrin receptor type 2 mutations (c.1100T>G, c.2008_9delAC, hereditary hemochromatosis type 3 analyzed by Kawabata). The patient received intravenous infusions of Laennec (672 mg/day, three times/week) or oral administration with Porcine (3.87 g/day) for 84 months as an alternative to repeated phlebotomy. At the end of the treatment period, serum ferritin level decreased to 428.4 ng/mL (below the baseline level of 536.8 ng/mL). Hemoglobin A1c levels also improved after treatment with the same or lower dose of insulin (8.8% before versus 6.8% after). Plural liver biopsies revealed remarkable improvements in the grade of iron deposition and fibrosis (F3 before versus F1 after) of the liver tissue.

**Conclusion:**

The discovery of hepcidin and its role in iron metabolism could lead to novel therapies for hereditary hemochromatosis. Laennec (parenteral) and Porcine (oral), which act as hepcidin inducers, actually improved iron overload in this hereditary hemochromatosis patient, without utilizing sequential phlebotomy. This suggests the possibility of not only improving the prognosis of hereditary hemochromatosis (types 1, 2, and 3) but also ameliorating complications, such as type 2 diabetes, liver fibrosis, and hypogonadism. Laennec and Porcine can completely replace continuous venesection in patients with venesection and may improve other iron-overloading disorders caused by hepcidin deficiency.

## Background

Iron is among the trace elements essential for the existence of all living organisms. It is required in an extensive diversity of metabolic processes, including Deoxyribonucleic acid (DNA) synthesis, oxygen transport, energy production, and innate immunity; in the activation of iron-containing enzymes, such as the cytochrome system in the mitochondria; and in the expression of other enzymes involved in the oxidation or reduction of biological substrates [1]. Excessively active cells need iron to maintain their metabolic vivacity because iron designates an appropriate chemical property for electron transfer, inducing biochemical interactions between many kinds of atoms, molecules, and chemical compounds. If inadequate iron exists in different ionic states, cells would lose their capacity for electron transport and oxidative energy metabolism. Elevated iron quantities can be extremely toxic due to the ability of iron to donate and accept electrons that catalyze the conversion of hydrogen peroxide into reactive oxygen species (ROS), which can cause unfavorable cell injuries and fatal organic damage [2]. As there is no known physiological mechanism for removing a large amount of iron appropriately and efficiently, even in severely iron-overloaded conditions, a crucial element in maintaining systemic iron homeostasis is effective communication among cells that absorb iron from the diet (duodenal enterocytes), use iron (mainly erythroid precursors), and store iron (hepatocytes and tissue macrophages). Therefore, when the iron intake exceeds cellular requirements, and storage capabilities are saturated, toxicity due to iron overload may occur [3]. Thus, the iron balance is maintained through sophisticated regulatory mechanisms.

The peptide hormone hepcidin interacts with the cellular iron exporter ferroportin (FPN) and is thus recognized as the principal regulator of systemic iron homeostasis [4]. Recent studies have established the importance of hepcidin in iron homeostasis as a negative regulator of iron release into the bloodstream by duodenal enterocytes and reticuloendothelial macrophages [5, 6]. Disturbances in the regulation of hepcidin are involved in the pathogenesis of several kinds of iron-related disorders, such as iron overload in hereditary hemochromatosis (HH) and nontransfused β-thalassemia; meanwhile, the overproduction of hepcidin is associated with the development of iron-restricted anemia, which is observed in patients with chronic kidney disease, chronic inflammatory diseases, some cancers, and inherited iron-refractory iron deficiency anemia [7]. In anemia of inflammation, hepcidin production is increased up to 100-fold, which may account for the characteristic sequestration of iron in macrophages [8]. As the human body does not have the capacity to deliberately increase the excretion of iron, untreated hemochromatosis will inevitably lead to bone and joint diseases, cirrhosis, liver cancer, diabetes, hypogonadism, impotence, depression, hypothyroidism, and premature death due to liver or heart failure [9]. In genetic or acquired iron overload, the hepatocytes become the major site of iron deposition, presumably because their iron uptake exceeds their capacity for physiological export [1]. Excess iron in the liver promotes steatohepatitis, liver fibrosis, cirrhosis, and hepatocellular carcinoma [10]. Iron absorption increases several fold in patients with iron deficiency and is partly suppressed when there is excessive iron storage.

Human hepcidin, a 25-amino acid peptide produced by hepatocytes, may be a new mediator of innate immunity and a long-sought iron-regulatory hormone. The discovery of hepcidin and elucidation of its role in iron metabolism made it possible to develop new therapies for hemochromatosis, anemia of inflammation, and other iron-related disorders [8]. Generally, hepcidin has a hyposideremic action, regulating iron metabolism in a sensitive manner. The regulation of hepatic synthesis of hepcidin is extremely complex and induces various signals and biological changes, some of which are indistinct. Indeed, hepcidin synthesis is stimulated by excessive iron uptake and inflammation, whereas it is repressed by iron deficiency and all pathological conditions that stimulate erythropoietic response (such as hemolysis, anemia, dyserythropoiesis, bleeding, and erythropoietin injections) [11]. In 2000, hepcidin was initially reported as an antimicrobial peptide [12]. In 2001, it was identified as the principal regulator of iron metabolism in humans, mainly decreasing iron absorption and increasing iron retention both in the macrophages of Reticuloendothelial system (RES) and Kupffer cells of the liver [13]. Thereafter, the identification of hepcidin as the master regulator of systemic iron homeostasis has enhanced our understanding of iron. Hepcidin has a distinct and essential role in controlling the dietary absorption of iron, its storage, and its release into the bloodstream. Hepcidin concentrations are strictly controlled, and their pathologic dysregulation leads to a number of human iron-related disorders. Our understanding of hepcidin regulation has rapidly increased, but numerous questions related to hepcidin pathobiology still need to be addressed [14].

Recent studies reported that hepcidin is deficient in patients with HH as a result of mutations in transferrin receptor type 2 (*TfR2*), *HFE*, or hemojuvelin (*Hjv*) genes [15]. Therefore, these genes must encode the regulators of hepcidin synthesis. Recent studies have also shown that these molecules form a hepcidin-regulating complex, possibly involving bone morphogenic proteins (BMPs) and their receptors [16]. However, how iron is sensed by these molecules and which signaling pathways they activate to regulate hepcidin in response to changes in iron concentrations still need to be elucidated further [8]. Hepcidin acts by binding to FPN, the sole cellular iron exporter, and induces its internalization and degradation [17]. As FPN is expressed in the duodenum, spleen, liver, and placenta, when hepcidin production increases, the level of FPN decreases, resulting in the inhibition of duodenal iron absorption, the release of recycled iron from macrophages, mobilization of iron stores from the liver, and the transfer of iron across the placenta [18]. Hepcidin may also limit bacterial proliferation by decreasing iron levels in the plasma and extracellular fluids. With regard to immune activity, hepcidin synthesis is highly induced by inflammatory signals, such as interleukin-6, thus playing a major role in the development of anemia associated with chronic inflammatory diseases [19]. Since the discovery of its hyposideremic action, considerable efforts have been made to explore the role of hepcidin in the management and regulation of iron. Almost all previous studies focused on the involvement of the liver as the major source of systemic hepcidin. However, interesting data have shown hepcidin production in several organs (kidney [20], macrophages [21], stomach [22], adipose tissue [23], and pancreas [24]), but the involvement of hepcidin produced in several peripheral organs in local and overall iron homeostasis remains unclear [11]. Serum hepcidin levels are mainly correlated with the actual levels of hepatic hepcidin expression [25], demonstrating that hepatic hepcidin is the key regulator of systemic iron balance. Data published by Kulaksiz *et al.* [24] concerning hepcidin expression in the pancreas demonstrated that hepcidin is produced in the pancreas of rats and humans. Further analysis showed that it was specifically localized in the β-cells of the islets of Langerhans. The *in vitro* experiments performed in this study demonstrated that the hepcidin expression in β-cells is directly regulated by iron. Iron is also important for maintaining normal insulin production and secretion. However, excessive levels of iron affect the β-cell function in hemochromatosis models [26, 27]. Phlebotomy (venesection therapy) is the standard treatment for patients with HH and has been used for more than 70 years. It is effective in reducing the morbidity and mortality rates among patients with HH [28]. Iron overload leads to tissue injury mainly through the production of ROS, which damages the cell membranes and organelles, resulting in cellular death [29]. Future hepcidin-based therapies might become a potential adjunct treatment for phlebotomy in both the induction and maintenance phases [29, 30].

In this case report, we present a newly developed “smart” treatment for HH utilizing placenta-derived drugs as “hepcidin inducers,” which have no apparent side effects and are more cost-effective (less than 250–300 USD/month in Japan) for clinical use. To confirm and clarify the fundamental background of this placenta-derived drug, we examined and analyzed the “hepcidin-inducing effect” of Laennec using primary liver cell cultures and HepG2 cells *in vitro*.

## Case presentation

**Fig. 1:**
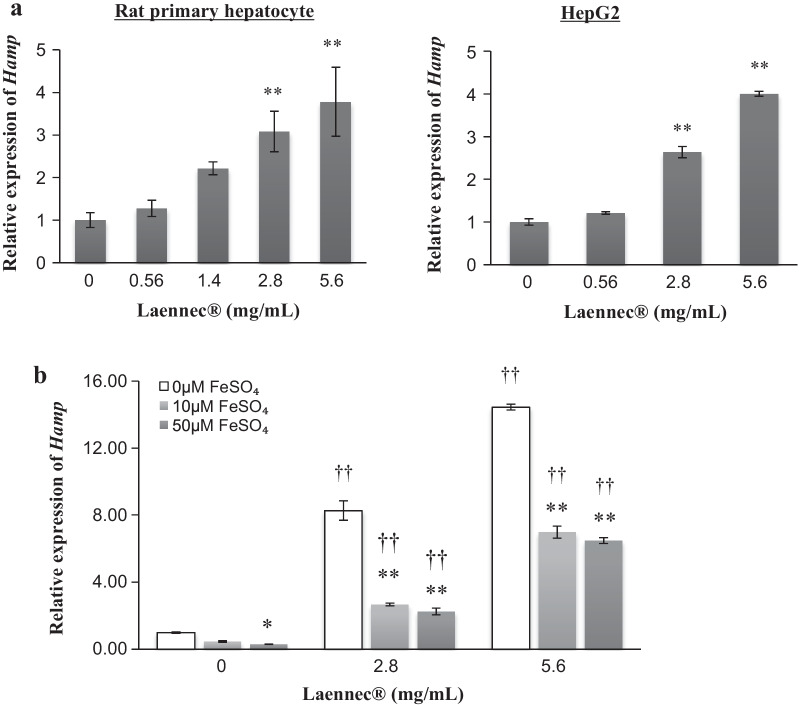
Laennec induces the expression of hepcidin mRNA in both rat primary hepatocytes and HepG2 cells.** a** The possibility of inducing hepcidin through the administration of Laennec was examined using rat primary hepatocytes and HepG2 cells as target cells *in vitro*. Laennec induces the expression of hepcidin mRNA both in rat primary hepatocytes and HepG2 cells in a dose-dependent manner. Results are expressed as mean ± SD (**p < 0.01 versus 0 mg/mL Laennec). **b** Dose dependency in inducing the expression of *Hamp* by treatment with Laennec in iron-rich environment in HepG2. In the iron-rich environment, although the magnitude of response in inducing hepcidin secretion is relatively attenuated, hepcidin mRNA secretion is also observed in a dose-dependent manner. Results are expressed as mean ± SD (*p < 0.05, **p < 0.01 versus 0 µM FeSO_4_; ^††^*p* < 0.01 versus 0 mg/mL Laennec

The possibility of inducing hepcidin expression through the administration of Laennec was examined using rat primary hepatocytes and HepG2 cells as target responders in vitro. Data in Fig. 1a show that Laennec induces hepcidin messenger Ribonucleic Acid (mRNA) expression, in both rat primary hepatocytes and HepG2 cells, in a dose-dependent manner; this suggests that in humans, the hepcidin-mediated action of Laennec could be one of the main processes involved in the regulation of iron metabolism and other iron-related pharmacological actions. Similar experiments were performed in an iron-rich medium (Fig. 1b). In this environment, although the magnitudes of response to induce hepcidin expression are relatively attenuated, hepcidin mRNA is typically expressed in a dose-dependent manner. This suggests that, in patients with hemochromatosis, cytotoxicity due to oxidative stress caused by high iron concentrations was controlled by administering Laennec, which stimulated the production of hepcidin. Laennec might induce hepcidin expression effectively and consistently, even in environments with high oxidative stress caused by iron overload.

**Fig. 2:**
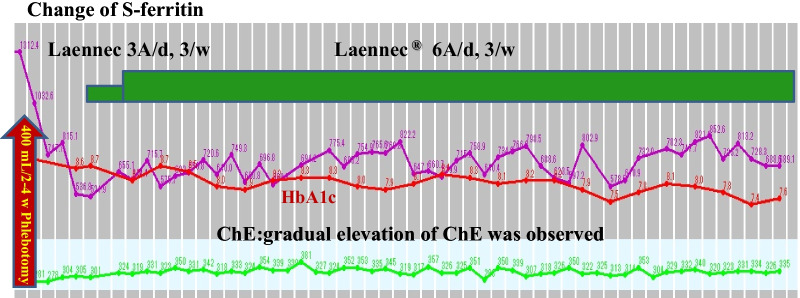
Hereditary hemochromatosis treated with Laennec. Laennec has been used for 24 months without phlebotomy. During this period, the serum ferritin elevation was only 150 ng/mL. The HbA1c level has also improved from 8.8% to 7.5% without increasing the dose of insulin. Approximately 20,800 mL (10,400 mg Fe) of blood was not collected

**Fig. 3:**
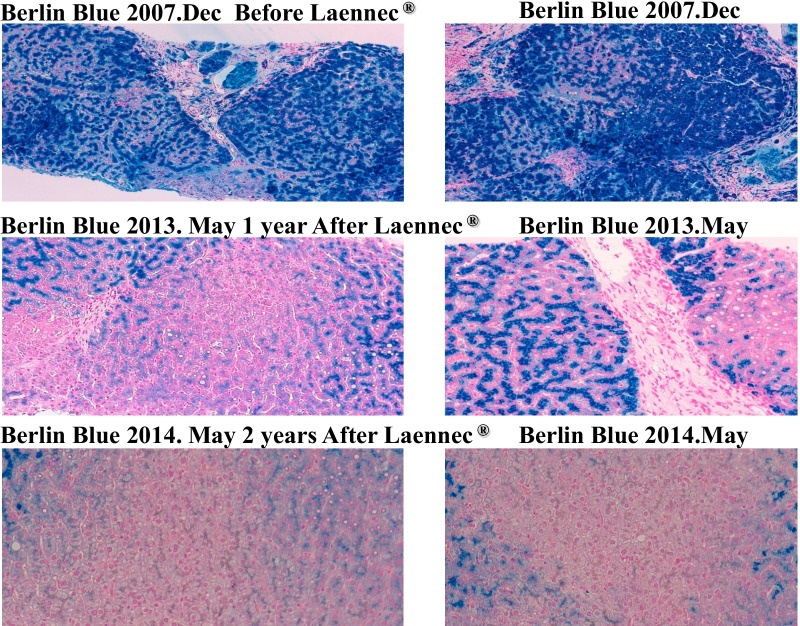
Pathological changes in hereditary hemochromatosis before and after Laennec treatment (2007/2013). During this period without phlebotomy, the histopathological evaluation revealed remarkable attenuation of iron deposition in the hepatocytes

**Fig. 4:**
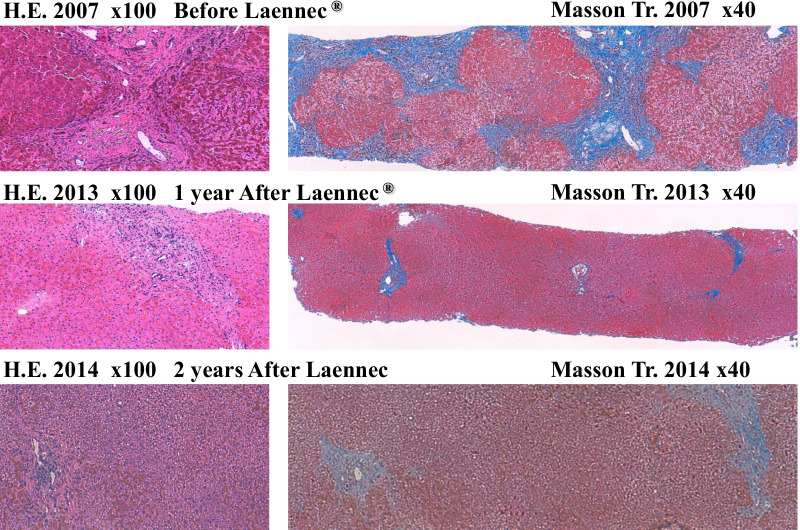
Pathological changes in hereditary hemochromatosis before and after Laennec treatment (2007/2013/2014). During this period without phlebotomy, the histopathological evaluation revealed remarkable attenuation of liver fibrosis

Laennec has been used for 24 months without phlebotomy (Fig. 2). During this period, the serum ferritin level increased to 150 ng/mL, whereas the hemoglobin A1c (HbA1c) level improved from 8.8% to 7.5% without increasing the dose of insulin. A total of 20,800 mL (10,400 mg Fe) of blood was not collected. If the patient refused to undergo phlebotomy and did not receive treatments for 2 years, the “estimated FT elevation” was 9776 ng/mL. However, the actual FT level only increased to 156 ng/mL, equivalent to 1.5% of the estimated FT elevation (Fig. 2). During the period without phlebotomy, histopathological evaluation revealed remarkable attenuation of iron deposition in the hepatocytes (Fig. 3) and a noticeable improvement in liver fibrosis (Fig. 4).

**Fig. 5:**
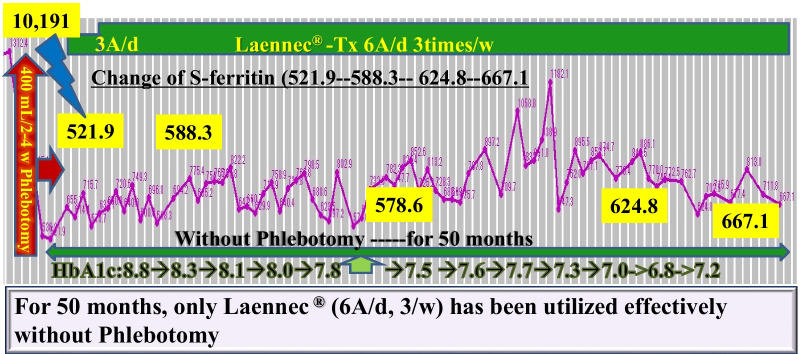
Hereditary hemochromatosis type 3 treated with Laennec. TfR2 mutation Laennec has been used for 50 months without phlebotomy. During this period, the serum ferritin elevation was only 146 ng/mL (0.78% of the estimated ferritin elevation)

**Fig. 6:**
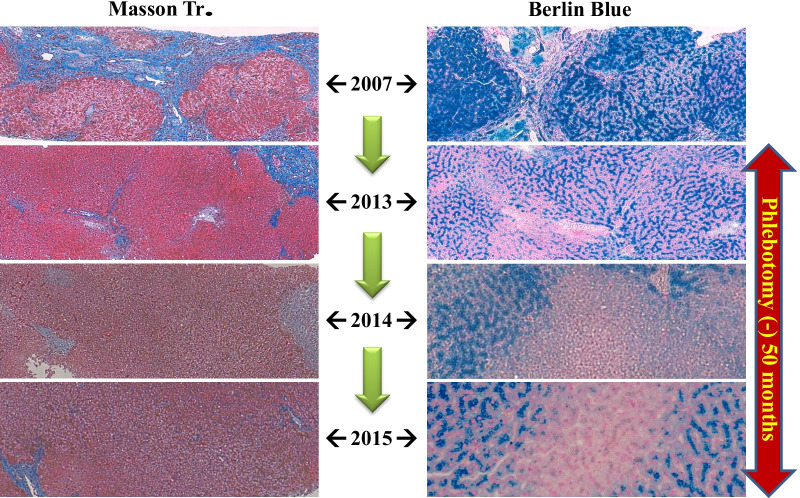
Pathological changes in hereditary hemochromatosis before and after Laennec treatment. After treatment with Laennec without phlebotomy for 50 months, the histological evaluation revealed a remarkable improvement of iron deposition and fibrosis

Laennec has been used for 50 months without phlebotomy (Fig. 5). During this period, the serum ferritin level only increased to 146 ng/mL, while the Glycosylateded Hemoglobin A1c (HbA1c) level improved from 8.8% to 6.8% without increasing the dose of insulin. A total of 39,600 mL (19,800 mg Fe) of blood was not collected. The actual ferritin level was only 146 ng/mL, which is equivalent to 0.78% of the estimated ferritin elevation (Fig. 5). Histopathological evaluation revealed further improvement in iron deposition and liver fibrosis (Fig. 6).

**Fig. 7:**
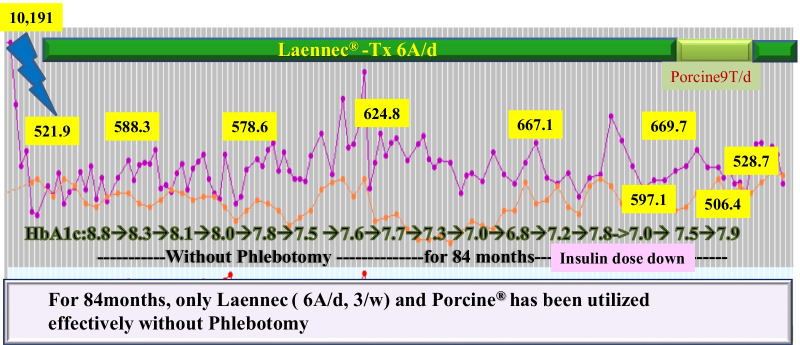
Hereditary hemochromatosis treated with Laennec and Porcine. Approximately 67,200 mL (33,600 mg Fe) of blood was not collected: estimated ferritin elevation, 31,584 ng/mL (47/100 mL). However, the actual ferritin level decreased (a decrease of 93.5 ng/mL compared with the baseline level); meanwhile, the HbA1c level improved from 8.8% to 6.8% without increasing the dose of insulin

**Fig. 8:**
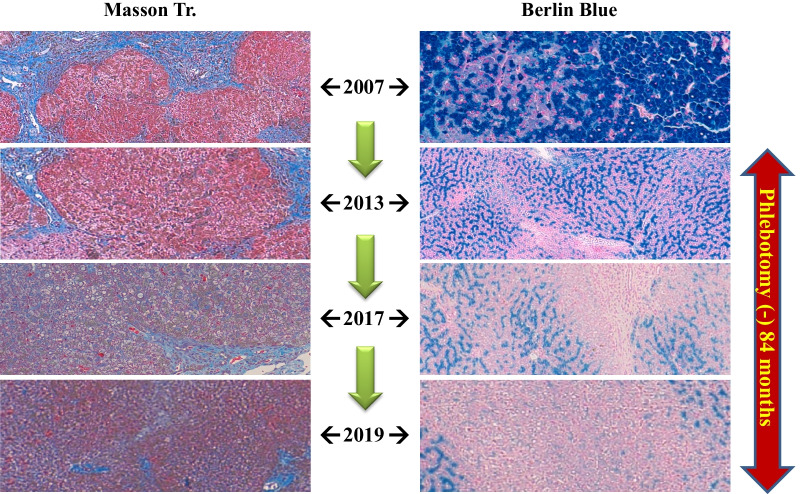
Pathological changes in hereditary hemochromatosis before and after Laennec treatment. After treatment with Laennec without phlebotomy for 84 months, the histological evaluation revealed a remarkable improvement of iron deposition and fibrosis

After treatment with Laennec without phlebotomy for 84 months (Fig. 7), the histopathological evaluation revealed a remarkable reduction in iron deposition and resolution of liver fibrosis. At the same time, the patients’ quality of life (QOL) significantly improved. Laennec can potentially enhance the iron loading condition without phlebotomy. During the treatment period, Porcine (a placenta-derived oral medicine) was replaced with Laennec, which was administered for 8 months; however, the efficacy remained the same (Figs. 7, 8). At the end of the treatment period, the serum Ferritin (FT) level decreased without phlebotomy (a total decrease of 93.5 ng/mL compared with the baseline level), whereas the HbA1c level consistently improved and ranged from 6.8% to 7.5%, despite the sustained reduction of insulin levels for 2–3 years.

This study is the first to describe the hepcidin-inducing effect of Laennec *in vitro* and *in vivo*. High hepcidin expression was associated with the regulation of iron observed in our HH case before Laennec treatment (hepcidin level before Laennec injection: 0.70 ng/mL; after Laennec injection: 3.40 ng/mL). In the present study, the efficacy of Laennec was possibly due to the hepcidin-inducing effect of this placenta-derived drug, which can completely substitute the phlebotomy treatment for HH patients.

## Discussion and conclusions

### Mode of action of Laennec and Porcine

Iron concentration in the body is rigorously regulated. The intracellular supply and storage of iron is mediated principally by three proteins: transferrin (Tf), TfR, and FT. Tf is a serum glycoprotein responsible for transporting bound iron from the absorbed organs to the peripheral tissues [31]. Furthermore, Tf, a component of an iron-sensing system, is essential for the maintenance of iron homeostasis by modulating hepcidin production [32]. Most cells modulate iron uptake by regulating the expression and dynamics of TfR. Regulation is mediated by intracellular iron levels and iron-responsive elements within the cell: mRNAs that are recognized by special iron regulatory proteins [31]. Hepcidin, a principal regulator of iron, is secreted into the bloodstream and interacts with enterocytes to adjust the rate of iron absorption [9, 33]. Bone morphogenetic protein (BMP)–SMAD (SMA and mothers against decapentaplegic homolog) signaling is modulated by various cofactors, including positive factors, such as *TfR2, HFE*, and *Hjv*, and negative factors, such as TMPRSS6 and soluble Hjv. The expression of BMP6 mRNA was recently shown to increase with iron loading in mice, suggesting that BMP6 is a signal that reflects the iron stores [34]. BMP–SMAD signaling is known to regulate hepcidin synthesis. BMPs are cytokines belonging to the TGF superfamily that exist primarily and abundantly in placental tissues. They play crucial roles in regulating cell proliferation, differentiation, and apoptosis throughout the development of each tissue [35]. Recent studies suggest that hepcidin, a peptide involved in iron homeostasis, is regulated by BMPs, apparently by binding to Hjv as a coreceptor and signaling through the SMAD4. The TfR2/HFE complex is also required for transcriptional regulation of hepcidin by the holo-Tf.

With the use of placenta-derived drugs as substitutes to phlebotomy, and based on the evidence from HAMP assays using HepG2 cells and primary hepatocyte cultures shown in Fig. 1a, b, one of the possible mechanisms and surmised sites of action inducing the expression of hepcidin by placenta-derived extracts might be the stimulation and/or acceleration of the BMP–SMAD–Hjv axis, which is located downstream of the intrahepatic iron-sensing system.

### Possibility of hepcidin-inducing treatment: efficacy on liver fibrosis and iron deposition

Hepatic fibrosis is a complicated dynamic process caused by hepatocyte death and activation of hepatic stellate cells (HSCs). Lipid peroxidation, including the generation of ROS, TGF-β, and TNF-α, is a cause of hepatic fibrosis. HSCs are regarded as the primary target cells that elicit an inflammatory response and produce extracellular matrix components. HSCs are activated by several factors, such as ROS, lipid peroxidation products [malonaldehyde and 4-hydroxynonenal], and TGF-β, which is released when adjacent cells, including hepatocytes, Kupffer cells, and endothelial cells, are injured by various types of disadvantageous pathognomonic abnormalities. The inflammatory generation of Reactive oxygen species (ROS) depends on the Fenton reaction catalyzed by redox-active metals, of which iron predominates. Iron overload causes oxidative stress and subsequent mitochondrial and DNA damage, lipid peroxidation, and protein modification, leading to the dysfunction of metabolically active inflated cells, such as cardiomyocytes, hepatocytes, pituitary cells, chondrocytes, and pancreatic β-cells [36]. In HFE-related HH, juvenile HH, and type 3 HH, iron is typically stored in periportal hepatocytes, with little or no iron stores in the Kupffer cells. As iron accumulation continues, the midzonal and centrilobular hepatocytes along with the biliary epithelium will progressively accumulate iron [37].

New data from a recent study suggest that impaired BMP signaling underlies hepcidin deficiency in HFE-related HH. Moreover, the inhibitory SMADs, SMAD6 and SMAD7, have been identified as potential disruptors of this signal and, hence, contribute to the pathogenesis of this disease [38]. Although it is classically regarded that phlebotomy cannot reverse liver cirrhosis, diabetes, or hypogonadism [39], our clinical data (Figs. 3, 4, 6, and 8) do not agree with these assumptions. By reducing iron-related ROS, which induce liver fibrosis and cause other organic injuries, the administration of Laennec (parenteral) and Porcine (oral) seems to treat these complications (diabetes and hypogonadism) in HH patients and other related pathological disorders (liver fibrosis and iron deposition in the hepatocytes).

### “A farewell to phlebotomy” in the treatment of iron-related disorders: disadvantages of venesection therapy

Phlebotomy (venesection therapy) is the standard treatment for patients with HH and has been used for more than 70 years. It is effective in reducing the morbidity and mortality rates among HH patients [28]. Iron-depleted HFE C282Y patients have extremely low serum hepcidin concentrations, reflecting the combined effects of HFE mutation and phlebotomy on hepcidin expression [40]. These patients demonstrated an exaggerated increase in the serum iron concentration upon oral iron loading. This observation indicates that reducing the hepcidin concentrations by phlebotomy in HH patients may exacerbate the underlying cause of iron overload, enhancing the excess release of iron into the circulation, with an ensuing “vicious cycle” that leads to the need for more frequent maintenance phlebotomies in HH patients [40]. Correspondingly, intestinal iron absorption is greatly increased in patients with hemochromatosis, even when the iron stores are depleted by phlebotomy. It is important to continue phlebotomies even if the iron stores are depleted to prevent the reaccumulation of iron. However, phlebotomy cannot reverse liver cirrhosis, diabetes, destructive arthritis, cardiomyopathy, or hypogonadism, which are the major complications of HH [39]. Although phlebotomy is specifically performed to remove excess “iron” in the blood, it can also reduce the amount of multiple nutrients, several kinds of essential hormones, and immune-related elements primarily contained in the blood of HH patients. Due to this inefficient and wasteful procedure, the QOL of HH patients tend to deteriorate, not only during the induction phase but also during the maintenance periods.

### Efficacy on T2DM and correlation between iron and glucose metabolism in iron-related chronic liver diseases

Persistent iron overload can generally lead to the development and progression of diabetes by lowering insulin production and promoting insulin resistance [41]. In addition to the apparent iron overload in hepatocytes, an increase in the body iron pool can also cause insulin resistance, metabolic syndrome, and gestational diabetes [42]. This assumption is empirically endorsed and based on the following observations: First, reduction of body iron pool by bloodletting or blood donation improves glycemic control and insulin resistance in patients with T2DM [43]; second, chelation therapy and blood donation lower the risk of diabetes in healthy individuals as well as in those with mildly increased body iron stores; and third, iron deficiency enhances insulin sensitivity and lowers the risk of diabetes [44]. Iron overload causes insulin deficiency by promoting pancreatic β-cell apoptosis. Because of their stringent dependence on mitochondrial glucose metabolism and their limited antioxidant capacity [45], β-cells are extremely susceptible to oxidative stress. Through their divalent metal transporter, pancreatic β-cells avariciously take up non-Tf-bound iron [46], which can promote oxidative stress by catalyzing the Fenton reaction. Diabetes is a one of the classic symptoms of HH, and some studies reported that the prevalence of diabetes in people with HH aged 45 years and older exceeds 20% [47]. Several studies have shown an association between hemochromatosis and T2DM [10, 48]. Elevated iron levels oxidize various biomolecules, such as nucleic acids, proteins, and lipids, which may contribute to the development of T2DM by decreasing insulin secretion from pancreatic β-cells, with concomitant increase in insulin resistance [3, 49]. With regard to the efficacy of bloodletting on T2DM, it is generally regarded that phlebotomy as a method used for achieving iron depletion does not improve diabetes control in all people with HFE-related hemochromatosis [50].

In the first 24 months of Laennec treatment (Fig. 2), the HbA1c level has also improved from 8.8% to 7.5% without increasing the dose of insulin, and has further improved from 8.8% to 6.8% for 50 months without changing the medications for diabetes treatment (Fig. 7). These results are presumably due to the additive efficacy of Laennec in reducing iron-originated ROS, enhancing the antiinflammatory action with concomitant improvement in liver fibrosis, and diminishing the iron deposition in the hepatocytes. Laennec was also administered in nonalcoholic steatohepatitis (NASH) patients with T2DM; treatment with Laennec significantly improved the T2DM, reduced the serum ferritin level, and decreased the iron deposition in the hepatocytes [51, 52]. The regulation of iron and glucose metabolism is possibly due to the pancreatic β-cells’ ability to co-release insulin and hepcidin. Even under pathophysiological conditions, there seemed to be a link between the secretion of insulin and that of hepcidin, since patients with HH not only exhibited a decrease in insulin secretion [53] but also a reduction in serum hepcidin levels [54]. As for the hepcidin expression in the pancreas, data published by Kulaksiz *et al.* [24] demonstrated that hepcidin is expressed in the pancreas of rats and humans. Further analysis showed that it was localized in the β-cells of the islets of Langerhans. In addition, the *in vitro* experiments performed in this study demonstrated that hepcidin expression in β-cells is directly regulated by iron. Iron is important for normal insulin secretion. However, excessive amounts of iron can affect the β-cell function in hemochromatosis models [26, 27, 55], causing iron accumulation in the islets, a reduction in insulin secretion, and an increase in the apoptosis of β-cells. By contrast, a decrease in the iron pool was shown to protect against diabetes and loss of β-cell function in an obese (ob/ob) mouse model [56]. These observations suggest that hepcidin produced by β-cells may be involved in the intrinsic regulation of pancreatic iron and glucose homeostasis [11]. Iron overload is a risk factor for diabetes. The link between iron and diabetes was first recognized in patients with HH and thalassemia, but high levels of dietary iron at the same time may enhance the risk of diabetes. Iron plays a direct and causal role in the pathogenesis of diabetes, mediated by both β-cell failure and insulin resistance. Iron also regulates metabolism in most tissues involved in fuel homeostasis, with adipocytes playing an iron-sensing role. The underlying molecular mechanisms mediating these effects are numerous and not completely understood, but include oxidative stress, modulation of adipokines, and intracellular signal transduction systems [33, 57]. Iron deposition also induces insulin resistance by inhibiting glucose uptake in fat and muscle tissues, and by reducing the capacity of the liver to extract insulin, which results in an abnormal increase in hepatic glucose production [58].

It seems reasonable to consider that one possible mechanism for the efficacy of Laennec in improving T2DM was the induction of the action of “local hepcidin” from pancreatic β-cells. However, further examinations are necessary to clarify the pharmacological dynamics of placenta-derived drugs.

### Efficacy of Laennec and Porcine on other iron-related diseases, especially on NASH complicated with T2DM

Serum ferritin is an independent predictor of histologic severity and advanced fibrosis in patients with nonalcoholic fatty liver disease (NAFLD). NAFLD is now recognized as a major cause of chronic liver diseases, including liver cirrhosis and hepatocellular carcinoma (HCC) in Western countries [47]. With the recent progress in the understanding of iron metabolism in patients with HH at the molecular level, accumulating evidence suggests a link between altered iron metabolism and NAFLD. In the last decade, many studies have found an intimate relationship between hepatic iron and NASH or its progression [3, 59, 60]. When iron accumulates, it promotes oxidative free radical reactions, which have harmful effects on multiple organs. In HH, the accumulation of iron in the liver, heart, and pancreas leads to cirrhosis, heart failure, and diabetes, respectively. Even mild iron overload might aggravate insulin resistance, diabetes, atherosclerosis, colonic neoplasia, and NAFLD [51, 52, 57, 59]. Increased oxidative stress is widely regarded as a key factor in the progression and deterioration of chronic liver diseases [49]. Recently, hepatic iron overload has attracted attention because excessive iron accumulation causes severe cellular dysfunction by facilitating the production of ROS, and the liver, which is the principal organ for iron storage, is readily damaged by iron-induced ROS. Moreover, this phenomenon has been frequently observed in patients with both hepatitis C virus infection and Non-alcoholic steatohepatitis (NASH) [49]. Patients with NAFLD frequently experience elevations in serum TS, SF, or both, and elevated serum iron markers may possibly indicate an iron overload [50]. Previous studies [51, 52, 59, 60] revealed that the administration of Laennec significantly improved T2DM complicated with NASH and other chronic liver diseases, which suggests the importance of iron regulation on insulin-resistant T2DM showing hyperferritinemia [51, 52, 59, 60]. A high level of Serum-Ferritin (SF) is a component of insulin resistance (IR). High ferritin levels in IR patients are mainly the result of a chronic inflammatory state induced by NASH and complicated with a metabolic syndrome [61]. Reciprocally, iron interferes with the action of insulin in the liver [62]. In addition, iron is a potent pro-oxidant that increases cellular oxidative stress, causing inhibition of insulin internalization and action, which results in hyperinsulinemia, IR, and abnormal β-cell function due to iron toxicity [59, 60, 63].

In our clinical experience, treatment with Laennec could improve, not only T2DM but also advanced liver fibrosis, even in patients with relatively progressed NASH [51, 52, 59, 60].

### Application of Laennec and Porcine as treatments for other iron-overloading diseases, such as β-thalassemia and myelodysplasia

Secondary hemochromatosis is usually caused by multiple blood transfusions in patients with hemolytic anemia, such as thalassemia, sickle cell anemia, and myelodysplasia syndrome (MDS). Iron first accumulates in the RES macrophages and is later transferred to the parenchymal cells. With frequent blood transfusions, iron may accumulate faster in patients with genetic hemochromatosis; iron overload often leads to severe cardiomyopathy and liver cirrhosis, limiting the prognosis of iron overload disorders. β-thalassemia is prevalent in Mediterranean countries, the Middle East, Central Asia, India, southern China, and the Far East, as well as in countries along the north coast of Africa and South America [64]. Therapy consists of iron chelators because erythroid disorders, such as β-thalassemia and MDS, are characterized by ineffective hematopoiesis and anemia, and are frequently accompanied by reduced expression of HAMP and followed by iron overload. The current standard of treatment for these disorders includes transfusion and iron chelation. Phlebotomies cannot be performed because of the underlying anemia [65]. However, iron chelators have serious side effects, and transfusions can aggravate iron overload [66]. The mainstay of therapy for β-thalassemia major is lifelong red cell transfusion to improve anemia and suppress ineffective erythropoiesis [67]. Increased erythropoiesis suppresses hepcidin production, resulting in a greater supply of iron from duodenal absorption and iron storage release, allowing sufficient iron to be available for hemoglobin synthesis. However, the molecular mechanisms mediating hepcidin suppression are not well understood [68]. In iron-loading anemia (β-thalassemia and congenital dyserythropoietic anemia), the suppressive effect of erythropoiesis on hepcidin production [69] and the resulting increase in dietary iron absorption can cause systemic iron overload and iron-mediated damage to the liver and myocardium, even without blood transfusions. However, whether treatment with exogenous hepcidin can correct the cause of iron-loading anemias needs to be further elucidated [1]. Patients with thalassemia intermedia experience iron overload even if they do not receive blood transfusions, and their urinary hepcidin levels are remarkably low despite high plasma Tf saturation and systemic iron overload [69]. With the increasing use of transfusion therapy, iron overload has become a major cause of morbidity and premature mortality. More recently, the effective treatment of iron overload by iron chelation has dramatically improved patient survival [70]. In patients with thalassemia, anemia induced by phlebotomy or hemolysis also suppresses the levels of hepatic hepcidin mRNA. Importantly, the suppressive effect of hemolytic anemia was observed even in iron-overloaded mice, suggesting that the suppression of hepcidin levels by anemia has a stronger effect than the stimulation of hepcidin expression by iron overload. This hierarchy of effects could explain why iron overload commonly develops in individuals with certain hemolytic disorders such as thalassemia. However, these observations do not explain why iron overload occurs more commonly in patients with intramedullary hemolysis than in those with peripheral hemolysis or nonhemolytic anemia [8]. In thalassemia intermedia, hepcidin therapy is expected to have beneficial effects by curbing the hyperabsorption of dietary iron. Hepcidin agonists could be useful even in patients with transfused thalassemia. Although intestinal iron absorption contributes less to the total iron load in these patients, hepcidin therapy may help in the period when endogenous hepcidin levels decrease and intestinal iron uptake increases. Hepcidin diagnostics and future therapeutic agonists may help in the management of patients with β-thalassemia [71]. Although these proof-of-principle studies show promise, no hepcidin therapies are yet available. Hepcidin has several characteristics that limit its potential use as a therapeutic agent. Due to its length and complex 4-disulfide structure, it is very expensive to produce.

Probably Laennec and Porcine, which can effectively induce the production of hepcidin, will improve the iron overload observed in thalassemia intermedia patients without any noticeable side effects. Placenta-derived extract Laennec and Porcine are effective drugs in this type of iron overload disorder. In Egypt, β-thalassemia is the most common genetically determined, chronic, hemolytic anemia, with an estimated carrier rate of 9–10.5% [72]. Currently, thalassemias are managed with chelation therapy, but exogenous Tf [73], exogenous hepcidin [74], or hepcidin signaling agonists [75] may be used as effective options in the near future. Our findings on the efficacy of Laennec and Porcine will help in the appropriate management of not only HH patients but also thalassemia (intermedia, trait, and major) and MDS patients.

This study is the first to present the possibility of applying the placenta-derived drugs Laennec and Porcine for the control of preferable and desirable erythropoiesis. β-thalassemia causes ineffective erythropoiesis and chronic anemia and is associated with iron overload from both multiple blood transfusions and increased iron absorption; the latter is mediated by suppression of the iron-regulatory hormone hepcidin [76]. In accordance with these backgrounds, we should seek to determine whether β-thalassemia major/intermediate/trait, with or without transfusion-mediated inhibition of erythropoiesis, will improve after treatment with Laennec and Porcine as a hepcidin inducer.

### Ideal treatment for HH and other iron-related diseases

#### Desirable future of HH therapeutics

Hepcidin-based therapies might, in the near future, become a potential adjunct treatment in place of phlebotomy. The possibility of using hepcidin is based on the pathognomonic decline in hepcidin levels in HH patients, which is responsible for the increased serum concentrations of iron and Tf saturation, and subsequently for iron overload [29, 30]. A low serum hepcidin level as the cause of hemochromatosis raises the possibility of using hepcidin analogs as treatments for patients with HH. However, the costs of parenteral peptide therapy should be compared with those of simple and low-cost phlebotomy therapy. For these reasons, oral iron chelators are not commonly used for the treatment of hemochromatosis [77]. However, phlebotomy cannot reverse liver cirrhosis, diabetes, destructive arthritis, cardiomyopathy, or hypogonadism [39]. Hepcidin replacement therapy could treat the underlying cause; thus, interest in the development of hepcidin therapeutics is growing [39]. The manuscript by Liu *et al.* published in *Haematologica*, reports the identification of novel compounds that can increase hepcidin expression in normal mice as well as in animals affected by hemochromatosis and β-thalassemia intermedia (or nontransfusion-dependent thalassemia) [78]. The iron-mediated control of hepcidin is achieved through at least two mechanisms. First, hepcidin senses the amount of intracellular iron in liver sinusoidal endothelial cells and responds by synthesizing BMP6 and other similar ligands belonging to the TGFβ-like family [79]. Second, hepcidin increases the intracellular concentration of iron, which leads to the secretion of BMP6 from these cells [79]. As a consequence, BMP6 binds and activates receptors that trigger the phosphorylation of the SMAD complex and stimulate hepcidin expression in hepatic cells [80]. To date, most of the compounds that increased the expression of hepcidin show a very specific activity (that is hepcidin mimetics or FPN inhibitors) [81, 82]. In general, these drugs belong to one of the four main categories: (i) hepcidin mimetics, (ii) hepcidin inducers, (iii) FPN inhibitors, and (iv) erythroferrone inhibitors. TMPRSS6 inhibitors can be defined as hepcidin inducers and/or BMP/SMAD pathway activators [80].

#### An optimum drug for treating iron overload

The ideal drug should be administered orally or injected subcutaneously very infrequently, and should have a long lifespan and prolonged activity. The drug should also show a large spectrum of activity, so that it can limit iron absorption in patients with nontransfusion-dependent thalassemia and HFE-related hemochromatosis, as well as in those patients with other conditions in which iron absorption is further increased (for example β-thalassemia major) or in which iron absorption needs to be further suppressed to achieve a significant benefit (as in polycythemia vera) [81, 82]. In addition, the drug should not cause any side effects, particularly under chronic administration conditions. Obviously, a low production cost would also be desirable. Furthermore, the drug should have a clear mechanism of action, if possible [80].

#### Hepcidin treatment

Many human diseases are not only associated with but are also modulated by alterations in hepcidin concentrations [4]. Hepcidin-targeted therapies may improve the outcomes of patients with iron disorders. Although no specific hepcidin therapies are currently available, several compounds are under development as hepcidin agonists or antagonists [83]. Hepcidin agonists could be useful for preventing iron overload caused by hepcidin deficiency, such as Hereditary hemochromatosis (HH), β-thalassemia, and other iron-loading anemias, and possibly some acquired forms of non-hemochromatotic iron-overload diseases [4]. Furthermore, hepcidin has either a primary or secondary role in insulin resistance, which is a characteristic of T2DM. However, it remains inconclusive whether serum hepcidin levels are an independent risk factor in the etiopathogenesis of T2DM. Thus, more experimental and clinical studies are needed to confirm the claim that hepcidin plays a role in T2DM [87].

These results, obtained from clinical trials conducted in patients with HH replacing phlebotomy with Laennec and Porcine treatment for more than 7 years, suggest the possibility that the corresponding drugs would be able to supervise hepatic iron metabolism through the transcriptional regulation of hepcidin. In summary, Laennec and Porcine are extremely promising treatment options and could be used in patients affected by iron-related disorders in which increased hepcidin expression is desirable. If proven safe and effective, their use in clinical practice will steadily increase.

In conclusion, our presented clinical course of HH treated by placenta-derived drugs Laennec and Porcine, as well as the fundamental data on hepcidin-inducing effects *in vitro* using the corresponding drugs, suggest that they are involved in the supervision of hepatic iron metabolism through transcriptional regulation of hepcidin. This study demonstrates the novel therapeutic potential of placenta-derived drugs Laennec and Porcine in improving hepatic fibrosis and iron deposition in hepatocytes as well as complicated diabetes and hypogonadism. In addition, by inducing preferable and appropriate amounts of hepcidin, placenta-derived drugs could improve the clinical course of NASH complicated with T2DM and hyperferritinemia by attenuating iron-induced oxidative stress and iron accumulation in both hepatocytes and pancreatic β-cells [76].

Based on this study, the possibility of replacing phlebotomy with placenta-derived drugs, Laennec and Porcine, was indicated through the pharmacological mechanism of inducing hepcidin production and suppressing iron-related oxidative stress [85, 86]. Furthermore, these results strongly suggest that Laennec and Porcine are quite effective not only for HH but also for other iron-loading diseases, such as β-thalassemia, MDS, NASH complicated with T2DM, and autoimmune liver disease [primary biliary cirrhosis and autoimmune hepatitis] [51, 52, 59, 60]. These are promising drugs that can suppress iron-induced oxidative injury as well as iron deposition in multiple organs, which will improve the prognosis of patients who developed iron-overloading disorders [76].

Optimistically, this will pave the way for “a farewell to phlebotomy” trial using iron removal strategies utilizing the placenta-derived drugs Laennec and Porcine. This will also open the way for preventing the progression of iron overloading liver diseases and their complications, including liver cirrhosis and HCC, by inducing the secretion of hepcidin and antiinflammatory effects through treatment with these placenta-derived drugs [3].

Further studies should confirm the role of iron overload and hyperferritinemia in patients with chronic liver diseases, including NASH, and the action of placenta-derived drugs.

To the best of our knowledge, the presented study is the first to examine the efficacy of Laennec and Porcine in removing excess iron in hepatocytes and other organs, and the modulatory effect of hepcidin on erythropoiesis in patients with HH. We found that “hepcidin-inducing treatment” with placenta-derived drugs could completely replace phlebotomy. Furthermore, their efficacy surpassed that of the venesection treatment in terms of improvements in liver fibrosis, diabetes, hypogonadism, and QOL of patients [76]. Thus, more experimental and clinical studies are needed to confirm the efficacy against other iron-related diseases [84].

If the hepcidin-inducing substance cannot be identified clearly, a conclusive evaluation on the exact mechanism underlying the induction of hepcidin secretion with the use of Laennec and Porcine cannot be proposed; however, more attention should be paid to improving the condition of the patient if this procedure is effective in a number of medical situations, judging from the historical evaluations of the corresponding drugs. At any rate, it does not seem possible to discontinue the application of “phlebotomy” if it is difficult to manage iron-overloading diseases, such as HH and other iron-related disorders. In addition, in some types of β-thalassemia (especially intermediate), inducing hepcidin by administering Laennec and Porcine will improve the iron-overloading condition in these patients without affecting the underlying cause of their hemolytic anemia. Furthermore, even in iron-loaded chronic liver diseases, such as NASH, type B/C viral hepatitis, autoimmune liver diseases (primary biliary cirrhosis (PBC) and autoimmune hepatitis (AIH)), and Liver Cirrhosis/Hepatocellular carcinoma (LC/HCC), the efficacy of removing iron from the liver will improve the prognosis of patients with these types of hepatic disorders.

In summary, the placenta-derived drugs Laennec and Porcine are very promising treatment options and can be used in patients affected by such disorders in which increased hepcidin expression is desirable and advantageous. If proven safe and effective, their use in clinical practice will steadily increase [80]. Similar to classical placenta drugs, Laennec and Porcine have also been used as “anti-aging” treatments and “skin care products.”

## Data Availability

All data generated or analyzed during this study are included in this article.

## Abbreviations

HH: Hereditary hemochromatosis
T2DM: Type 2 diabetes mellitus
SF: Serum ferritin
TfR2: Transferrin receptor type 2
ROS: Reactive oxygen species
FPN: Ferroportin
Hjv: Hemojuvelin
BMP: Bone morphogenic proteins
HbA1c: Hemoglobin subunit alpha
FT: Ferritin
Tf: Transferrin
SMAD: SMA and mothers against decapentaplegic homolog
HSCs: Hepatic stellate cells
DMT: Divalent metal transporter
NAFLD: Nonalcoholic fatty liver disease
HCC: Hepatocellular carcinoma
IR: Insulin resistant
ED: Erection disturbance
